# Emerging Trends in Lung Cancer Presentation at a Leading Tertiary Oncology Center and the Need for Lung Cancer Screening in Pakistan

**DOI:** 10.7759/cureus.70381

**Published:** 2024-09-28

**Authors:** Syed Murtaza Hassan Kazmi, Asif Masood, Sumaira Gulzar, Azhar Shafi

**Affiliations:** 1 Department of Pulmonology, Shifa International Hospital Islamabad, Islamabad, PAK; 2 Department of Radiation Oncology, Shifa International Hospital Islamabad, Islamabad, PAK; 3 Clinical Research Center, Shifa International Hospital Islamabad, Islamabad, PAK; 4 Department of Medical Oncology, Shifa International Hospital Islamabad, Islamabad, PAK

**Keywords:** cancer staging, demographics, histological types, lung cancer, pakistan, retrospective study, symptoms

## Abstract

Background

Lung cancer is a significant global health concern, with Pakistan lacking a national cancer registry.

Methods

A retrospective study analyzed 345 lung cancer patients at Shifa International Hospital from 2018 to 2020. Demographics, symptoms, cancer stage, site, and histological types were assessed using descriptive statistics and Pearson’s chi-square test.

Results

Most patients were male (267, 77.4%) with a mean age of 45-75 years. Common symptoms included patients with cough (168, 49%), blood in sputum (114, 33%), and chest pain (120, 35%). Most cases presented at advanced stages (81% at stage 4). Adenocarcinoma (131, 38%) was the most common histological type, with significant differences noted between smokers and non-smokers.

Conclusion

Gender disparities exist in lung cancer incidence, with a higher proportion of male patients. Both smokers and non-smokers are affected, emphasizing the need for comprehensive screening. Early detection and intervention strategies are crucial, especially considering the advanced stage of presentation. Tailored approaches considering histological types are essential for effective management. This study underscores the urgency of implementing screening programs and raising awareness to mitigate the burden of lung cancer in Pakistan.

## Introduction

Cancer is a leading cause of death worldwide, with lung cancer ranking the second most common cancer all over the world after breast cancer sitting at the top, according to the GLOBOCAN and the International Agency for Research on Cancer (IARC) 2020 [1]. In 2020, 1.80 million people died of lung cancer worldwide, according to the World Health Organization (WHO) [2]. Unfortunately, Pakistan does not have a national cancer registry center, making it difficult to track the occurrence of different types of cancer on an annual basis. However, some of the cities have cancer registry centers, such as the Punjab Cancer Registry Lahore, Karachi Cancer Registry, Pakistan Atomic Energy Commission Cancer Registry (PAEC) (Islamabad), Shifa International Hospital Cancer Registry (Islamabad), Armed Forces Institute of Pathology (Rawalpindi), and Nishtar Medical University Cancer Registry (Multan) [3]. Recently, data were collected from these centers to publish a report on cancer from 2015 to 2019 at the national level, which reported lung cancer as the fifth most commonly occurring cancer (3.69%) in both sexes along with all age groups combined [4].

The management and prognosis of lung cancer vary depending on the histological classification and initial stage of cancer presentation. Lung cancer is mainly divided into two groups: small-cell lung cancer (SCLC) and non-small cell lung cancer (NSCLC) [5]. NSCLC develops at a slower rate than SCLC. NSCLC is further classified based on histopathological characteristics into squamous cell carcinoma, adenocarcinoma, and large cell carcinoma [6]. Adenocarcinoma occurs in the mucous-secreting glandular cells present in the lungs. It usually occurs in the outer part of the cell lining of the lungs and the bronchoalveolar lining of the airways [7]. Squamous cells that are responsible for the formation of the inner layer of the airways could transform into squamous cell carcinoma. Large cell carcinoma, when compared to squamous cell and adenocarcinoma, has a faster growth rate than both and it can be found in the lung at any site [8]. SCLC is a small aggressive kind of tumor that has a rapid growth fraction and is hard to recognize [9].

TNM (tumor, node, metastasis) staging system for lung cancer is developed by the American Joint Commission on Cancer (AJCC) and the Union for International Cancer Control (UICC) [10]. The characterization of lung cancer according to the TNM system classification depends upon the extent of the tumor, involvement of lymph nodes, and presence of distant metastasis. The TNM staging system of cancer to some extent is determined by the medical history of the patient, their physical examination, and the imaging and pathology reports generated by radiologists and pathologists, respectively [11].

Tobacco smoking, a family history of the disease, genetic polymorphism, diet and alcohol consumption, exposure to ionizing radiation, asbestos, and certain metals such as cadmium, copper, and arsenic are considered to be among the major contributing factors that lead to a higher risk for development of lung cancer [12].

Persistent cough turns out to be the most prominent symptom of lung cancer along with a wide range of symptoms, including shortness of breath, coughing up blood, chest pain, loss of appetite, and weight loss observed commonly in lung cancer patients. Chest X-rays are sometimes the investigation that leads to the identification of lung cancer in many patients who were undiagnosed earlier [13]. Poor survival rates and high fatality rates have been linked to undiagnosed early-stage lung cancer around the globe.

The purpose of the present study is to investigate patterns in the demographics, features, histological subtypes, and stages of lung cancer presentation at a tertiary oncology center.

## Materials and methods

Study design

This study utilizes a retrospective observational design, reviewing case records to analyze emerging trends in lung cancer presentation. Additionally, it aims to assess the need for lung cancer screening in Pakistan based on the collected data.

Study site and duration

The study was conducted at Shifa International Hospital, Islamabad, from 2018 to 2020.

Data sources

The primary data sources include patient records and hospital databases. Data were extracted from the electronic health records (EHR) of 345 lung cancer patients diagnosed and treated at Shifa International Hospital. Supplementary data from hospital databases provided additional information on patient demographics, clinical presentations, and treatment outcomes.

Inclusion and exclusion criteria

Inclusion criteria for the study were patients diagnosed with primary lung cancer between January 2018 and December 2020, those who have undergone diagnostic imaging (CT scans, X-rays) and histopathological confirmation of lung cancer, and patients with complete medical records available for demographic, clinical, and treatment details. Exclusion criteria included patients with secondary/metastatic lung cancer and those with incomplete medical records.

Data collection

Data collection encompassed demographic variables such as age, gender, smoking history (current, former, non-smoker), family history of lung cancer, and presenting symptoms including cough, chest pain, and hemoptysis. Clinical variables included cancer stage classified by the AJCC staging method using the TNM system for tumor size, lymph node involvement, and metastasis. Lung cancer types were categorized into SCLC and NSCLC, which included adenocarcinomas, squamous cell carcinomas, and large cell carcinomas.

Data analysis

Statistical analysis employed IBM SPSS V.21 (IBM Corp., Armonk, NY) and Microsoft Excel 2016 (Microsoft Corporation, Redmond, WA), utilizing descriptive statistics to summarize demographic variables and Pearson’s chi-square test to assess differences in categorical variables between smokers and non-smokers with lung cancer.

Ethical considerations

Informed consent was waived due to the retrospective nature of the study. Ethical approval was obtained from the institutional review board (IRB) of Shifa International Hospital (REF #049-22). According to local regulations and institutional policies, patient confidentiality and data protection will be ensured.

## Results

A total of 345 patients with lung cancer were included in the study, of which 111 (32%) patients were smokers and 234 (68%) were non-smokers. Of the 111 (32%) smokers, only three (2.8%) were females and 108 (97.2%) were males. Similarly, among 234 (68%) non-smokers, 75 (32.1%) were female and 159 (67.9%) were male. Overall, 267 (77.4%) males and 78 (22.6%) females were included in the study, with a higher proportion of men than females (Table 1). A chi-square test was conducted to evaluate the association between gender and smoking status among lung cancer patients. The test revealed a significant association between gender and smoking status (chi-square statistic = 37.0618, p < 0.00001), indicating that smoking was significantly more prevalent among male patients than female patients.

**Table 1:** Gender distribution.

Gender	Smoker		Total
	Smoker	Non-smoker	
Male	108 (97.2%)	159 (67.9%)	267 (77.4%)
Female	3 (2.8%)	75 (32.1%)	78 (22.6%)
Total	111 (32%)	234 (68%)	345 (100%)

**Figure 1:**
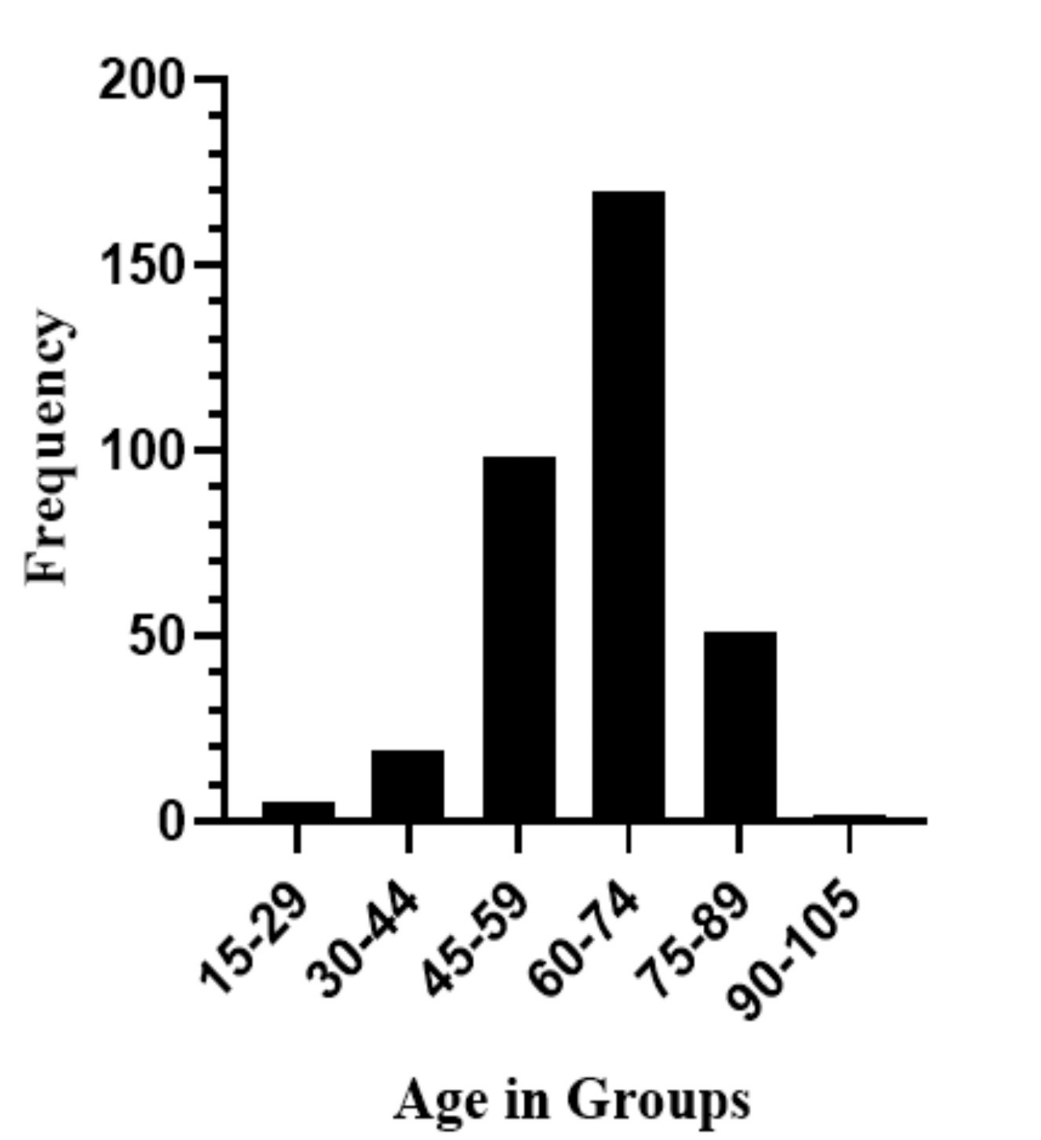
Age group distribution.

The ages of the patients ranged from 15 to 105 years, with most patients falling between the age bracket of 45 and 75 years (Figure 1).

The data on symptoms among lung cancer patients show no significant difference between smokers and non-smokers overall, with a p-value of 0.774. Cough was the most common symptom, reported by 168 (49%) patients, with non-smokers (110, 65%) reporting it more frequently than smokers (58, 35%). Blood in sputum was reported by 114 (33%) patients, again more common in non-smokers (68, 60%) compared to smokers (46, 40%). Chest pain affected 120 (35%) of the patients, with a higher prevalence among non-smokers (75, 63%) than smokers (45, 38%). Family history of lung cancer was noted in 49 (14%) of patients, with non-smokers (32, 65%) more likely to report it than smokers (17, 35%) (Table 2).

**Table 2:** Lung cancer symptoms and positive family history. In the total column, the percentages are out of 345 patients. The percentages in smoker and non-smoker groups are calculated by dividing the total number of smokers with that symptom by the total number of patients with that symptom.

Symptoms	Total		Smoker		Non-smoker		P-value
	N	%	N	%	N	%	0.774
Cough	168/345	49%	58/168	35%	110/168	65%	
Blood in sputum	114/345	33%	46/114	40%	68/114	60%	
Chest pain	120/345	35%	45/120	38%	75/120	63%	
Family history	49/345	14%	17/49	35%	32/49	65%	

The data comparing lung cancer morphologies among smokers and non-smokers revealed significant differences across the morphologies, with an overall p-value of <0.001 indicating a statistically significant difference in the distribution of lung cancer types between smokers and non-smokers. Adenocarcinoma is notably more prevalent among non-smokers (104, 44%) compared to smokers (27, 24%). Conversely, squamous carcinoma is more common in smokers (38, 34%) than in non-smokers (43, 18%). Small cell carcinoma also shows a higher prevalence in smokers (25, 23%) compared to non-smokers (34, 15%). Other morphologies such as neuroendocrine carcinoma, pseudosarcomatous carcinoma, and carcinoma not otherwise specified (NOS) exhibit similar distributions between the two groups, with no marked differences (Table 3).

**Table 3:** Comparison of lung cancer morphologies.

Morphology	Total		Smoker (N = 111)		Non-smoker (N = 234)		P-value
	N	%	N	%	N	%	
Adenocarcinoma	131	38.0%	27	24%	104	44%	<0.001
Squamous carcinoma	81	23.4%	38	34%	43	18%	
Small cell carcinoma	59	17.1%	25	23%	34	15%	
Neuroendocrine carcinoma	4	1%	1	1%	3	1%	
Pseudosarcomatous carcinoma	5	1.4%	1	1%	4	2%	
Carcinoma not otherwise specified	40	12%	16	14%	24	10%	
Others	25	7%	3	3%	22	9%	

Table 4 presents the distribution of various lung cancer morphologies across different bronchopulmonary segments in smokers and non-smokers. Adenocarcinoma is most prevalent in non-smokers within the upper lobe, while squamous cell carcinoma is more common among smokers in the lower lobe. Other carcinomas show varying distributions across different lobes and smoking statuses (Table 4).

**Table 4:** Distribution of lung cancer morphologies across different bronchopulmonary segments based on smoking status.

Morphology	Main bronchus (n = 25)		Upper lobe (n = 113)		Middle lobe (n = 11)		Lower lobe (n = 71)		Overlapping lesion (n = 6)	
-	Smoker	Non-smoker	Smoker	Non-smoker	Smoker	Non-smoker	Smoker	Non-smoker	Smoker	Non-smoker
Adenocarcinoma	1 (4%)	3 (12%)	9 (7.9%)	30 (26.5%)	-	5 (45.5%)	7 (9.85%)	18 (25.3%)	2 (33.3%)	1 (16.6%)
Squamous cell carcinoma	6 (24%)	5 (20%)	14 (12.4%)	24 (21.2%)	-	1 (9%)	13 (18.3%)	7 (9.85%)	-	-
Small cell carcinoma	4 (16%)	1 (4%)	7 (6%)	7 (6%)	-	1 (9%)	6 (8.45%)	4 (5.63%)	-	1 (16.6%)
Neuroendocrine carcinoma	-	1 (4%)	-	-	-	-	-	-	-	1 (16.6%)
Pseudosarcomatous carcinoma	1 (4%)	1 (4%)	-	1 (0.8%)	-	-	-	1 (1.4%)	-	-
Carcinoma not otherwise specified	1 (4%)	-	7 (6%)	9 (7.9%)	-	2 (18.2%)	3 (4.22%)	3 (4.2%)	1 (16.6%)	-
Others	-	1 (4%)	1 (0.8%)	4 (3.5%)	-	2 (18.2%)	1 (1.4%)	8 (11.2%)	-	-
Total	13 (52%)	12 (48%)	38 (33.6%)	75 (66.4%)	-	11 (100%)	30 (42.2%)	41 (57.7%)	3 (50%)	3 (50%)

## Discussion

This study was primarily designed to investigate the emerging trends in lung cancer presentation at Shifa International Hospital in Islamabad between 2018 and 2020. We analyzed the data of 345 patients diagnosed with lung cancer, taking into consideration the various demographic features, symptoms, cancer stage, site, and histological types. The findings of our analysis along with their significance in relation to lung cancer in Pakistan are thoroughly addressed in this section.

The analysis revealed a gender imbalance in lung cancer, with a higher proportion of male patients (78%) than female patients (22%). This variation is strongly in alliance with the global pattern of lung cancer incidence, which shows that men traditionally had a higher prevalence of the disease in comparison to women [14]. However, a substantial number of women in Pakistan are also affected by lung cancer, highlighting the importance of tailored gender-specific strategies for lung cancer screening and awareness [15,16].

The distribution of smokers and non-smokers in this study was 32% and 68%, respectively. This figure highlights that a significant proportion of lung cancer cases happen to target non-smokers, pinpointing that there is a need for comprehensive screening programs that target both smokers and non-smokers [17-19]. Furthermore, our data indicate a higher prevalence of lung cancer in the lower lobe and main bronchus among smokers, which is consistent with the well-established fact of association between smoking and lung cancer risk [20].

The most common symptoms experienced by the patients enrolled in the study were coughing, the presence of blood in sputum, and chest pain, with no significant variations observed between smokers and non-smokers. This information is suggestive of the fact that these symptoms can be indicative of lung cancer, regardless of smoking history, emphasizing the importance of early symptom recognition and timely diagnostic evaluation [13,21].

A significant majority (81%) of patients in our study were diagnosed at stage 4, highlighting the advanced-stage presentation of lung cancer in Pakistan. This underscores the urgent need for the implementation of effective lung cancer screening programs to enable early detection when the disease is more treatable. The common histological type was adenocarcinoma, followed by squamous cell carcinoma, and small cell carcinoma. Interestingly, the distribution of the histological types varied significantly between smokers and non-smokers. Adenocarcinoma was more prevalent among non-smokers, whereas squamous cell carcinoma was more common among smokers. This difference in the histological type of cancer may have consequences for treatment strategies and targeted therapies.

The study also highlights the need for the implementation of lung cancer screening programs in Pakistan, particularly for high-risk populations, including smokers and individuals with a family history of lung cancer. Although this retrospective observational study does not directly evaluate the impact of screening, the high proportion of late-stage diagnoses strongly supports the necessity of such programs to improve early detection rates and patient outcomes.

The limitations of our study are its retrospective nature and the fact that it was conducted at a single center. Furthermore, the data presented in this study may not fully represent the broader Pakistani population as a multicentered approach was not opted, rather it was only limited to patients at the Shifa International Hospital.

Our research conclusively highlights the need and importance of heightened awareness, early detection, and intervention strategies for lung cancer in Pakistan. The data unveil the gender disparities, highlight the substantial burden of advanced-stage disease, and stress the significance of considering both smokers and non-smokers in screening and prevention efforts to decrease the burden of disease. Lung cancer remains a daunting health challenge in Pakistan, and tackling it effectively demands a multifaceted approach that involves healthcare providers, policymakers, and the community all on board for effective preventive measures. By implementing comprehensive screening programs and disseminating information regarding the risk factors associated with lung cancer, we hope to reduce the impact of this disease in the future.

## Conclusions

In conclusion, this study provides strong evidence of the need for heightened awareness, early detection, and intervention strategies for lung cancer in Pakistan. The significant association between gender and smoking status, with a higher prevalence of smoking among male patients, underscores the importance of gender-specific approaches in lung cancer screening and prevention efforts. Additionally, the high proportion of late-stage diagnoses (81% at stage 4) further emphasizes the need for comprehensive screening programs targeting both smokers and non-smokers. Moreover, the variation in histological types between these groups emphasizes the necessity of considering individualized treatment strategies. By implementing effective screening programs and disseminating information on risk factors, we aim to mitigate the impact of lung cancer in Pakistan.
